# Natura 2000 Areas, Road, Railway, Water, and Ecological Networks May Provide Pathways for Biological Invasion: A Country Scale Analysis

**DOI:** 10.3390/plants10122670

**Published:** 2021-12-04

**Authors:** Péter Szilassi, Anna Soóky, Zoltán Bátori, Alida Anna Hábenczyus, Kata Frei, Csaba Tölgyesi, Boudewijn van Leeuwen, Zalán Tobak, Nándor Csikós

**Affiliations:** 1 Department of Geoinformatics, Physical and Environmental Geography, University of Szeged, Egyetem utca 2, H-6722 Szeged, Hungary; leeuwen@geo.u-szeged.hu (B.v.L.); tobak@geo.u-szeged.hu (Z.T.); csntact@gmail.com (N.C.); 2 Department of Ecology, University of Szeged, Közép fasor 52, H-6726 Szeged, Hungary; sookyanna001@gmail.com (A.S.); zbatory@gmail.com (Z.B.); alidaanna@gmail.com (A.A.H.); freikata98@gmail.com (K.F.); festuca7@yahoo.com (C.T.)

**Keywords:** LUCAS database, invasive plants, linear transport infrastructure, Natura 2000 areas, ecological network, blue infrastructure

## Abstract

Invasive species are a major threat to biodiversity worldwide. Controlling their rapid spread can only be effective if we consider the geographical factors that influence their occurrence. For instance, roads, railway networks, green and blue infrastructure, and elements of ecological networks (e.g., ecological corridors) can facilitate the spread of invasive species. In our study, we mapped the occurrence of five invasive plant taxa (tree of heaven, common milkweed, Russian olive, black locust, and goldenrods) in Hungary, using field photos from the EUROSTAT Land Use and Coverage Area Frame Survey (LUCAS) database from the year 2015. Species point occurrence data were compared with the spatial characteristics of linear transport infrastructure and with the green and blue infrastructure. We found that the occurrence of tree of heaven and Russian olive was strongly related to the road and railway network. The average Euclidean distance of LUCAS points infected with these species from railway embankments and roads was much smaller than that of uninfected points. However, black locust and goldenrods were more common only along the road network. According to our results, the occurrence of some investigated invasive plants was over-represented in the HEN and within Natura 2000 areas of Hungary compared to non-infected points. Our results may provide important information for predicting the rate of invasion and for applying targeted management within the HEN, and Natura 2000 protected areas.

## 1. Introduction

Globalisation is making biological invasions more prevalent. Nowadays, biological invasions and their negative ecological consequences are considered one of the major challenges for nature conservation [1,2]. According to a survey conducted by Genovesi and Monaco [3] in 21 European countries, the most serious threats to biodiversity are natural and semi-natural habitat loss and landscape fragmentation, followed by biological invasion. According to a questionnaire survey of Csiszár et al. [4], the spread of invasive species is also the most significant conservation problem of the national parks and other protected areas of Hungary.

Native species and natural communities are threatened by invasive species, mostly originating from other continents, spontaneously or deliberately introduced, causing a loss of biodiversity [5,6]. Invasive plant species can change species interactions, profoundly modifying the structure of entire food webs [7,8]. In addition, some invasive species (e.g., *Ambrosia artemisiifolia* and *Solidago* spp.) have harmful pollen and therefore pose a health risk, while others (e.g., *Robinia pseudoacacia*) increase soil nitrogen content, causing soil degradation and enabling weeds to germinate without competition [2,9]. Invasive tree species, such as *Elaeagnus angustifolia*, *Robinia pseudoacacia* and *Ailanthus altissima*, shade the herb layer, thus altering the microclimate of the area [10]. They can also cause degradation of the soil, leading to loss of soil fertility and the formation of soil debris [1,5,11,12].

According to Mihály and Botta-Dukát [13], three percent of the Hungarian vascular flora of about 2400 species belongs to adventive weed species, most of which are also sources of invasion. In Europe, a list of 11,778 taxa of invasive species has been compiled in the DAISIE and EASIN projects [11,14]. The European Union’s list of the most harmful invasive species includes 24 terrestrial plant species [15]. Among these species, the anthropogenic causes of the spread of the five most common invasive species in Hungary were investigated, with a particular focus on their distribution in relation to transport, green and blue infrastructure. 

In order to control invasive plants, it is of paramount importance to understand the environmental and anthropogenic factors that determine the occurrence and spread of invasive species. Roads, railway networks, green and blue infrastructure can facilitate the spread of certain invasive plants [16,17,18,19,20,21,22,23]. The Hungarian Ecological Network (HEN) was originally designed to facilitate the migration of native species between habitats of high conservation value [24]. In many cases, ecological corridors not only provide a pathway for indigenous species, but also for invasive species [25]. Therefore, they may facilitate invasion of natural and semi-natural habitat patches.

In this study, we mapped the occurrence of five widespread invasive plant taxa in Hungary: common milkweed (*Asclepias syriaca* L., Apocynaceae), tree of heaven (*Ailanthus altissima* (Mill.) Swingle, Simaroubaceae), Russian olive (*Elaeagnus angustifolia* L., Elaeagnaceae), black locust (*Robinia pseudoacacia* L., Fabaceae) and goldenrods (*Solidago canadensis L.* and *Solidago gigantea*, Aiton, *Solidago* spp., Asteraceae hereafter) by visual interpretation of the 2015 field survey source photos taken as part of the EU Statistical Office (EUROSTAT) Land Use and Coverage Area Frame Survey (LUCAS) database on a national scale throughout Hungary in 2015 [26]. The Landscape Ecological Vegetation Database and Map of Hungary showed the distribution of these species in 2006, but so far there has not been a new country scale database developed in this topic. In our previous studies, we have shown that the LUCAS database field photographs can be a useful database for mapping the occurrence of invasive plant species, identifying infection hotspots, and monitoring their spread [27]. Here, we analysed the GIS database containing LUCAS field survey-based point occurrence data of the five plants, which we compared with the spatial characteristics of the transportation infrastructure and the green and blue infrastructure (water and ecological networks, respectively). In our research, we created the spatial distribution map of five common invasive plant taxa of Europe, and based on this map we aimed to answer the following questions:

- How can point-based digital vegetation occurrence data be used to analyse the geographical context of biological invasions?
- What is the relative importance of road and railway networks in the spread of the studied invasive species?
- What is the role of the HEN and Natura 2000 areas in the spread of the studied invasive species?

## 2. Materials and Methods

### 2.1. Study Area

Hungary is a Central-European country with high soil productivity due to the large area of Chernozem soils and a humid continental climate with an average annual temperature of 10.5 °C and an average annual precipitation of 550 mm [28]. Arable land is the dominant land use type (about 50%) in lowlands, such as the Great Hungarian Plain, while the proportion of forests is relatively high in hilly and mountainous areas. Many natural and semi-natural habitat types (mainly temperate forests and grasslands) occur within national protected areas or are part of the Natura 2000 network of the European Union. However, the invasion of invasive species has seriously damaged many of these natural and semi-natural habitats. According to the Landscape Ecological Vegetation Database and Map of Hungary (MÉTA database), in 2006, 13.1% of the total natural habitats was invaded by invasive species [29].

All of the five investigated invasive plant taxa are common in Hungary and Europe [15]. Hungary is one of the countries most affected by invasive species in Europe [30].

### 2.2. Digital Databases

**Figure 1:**
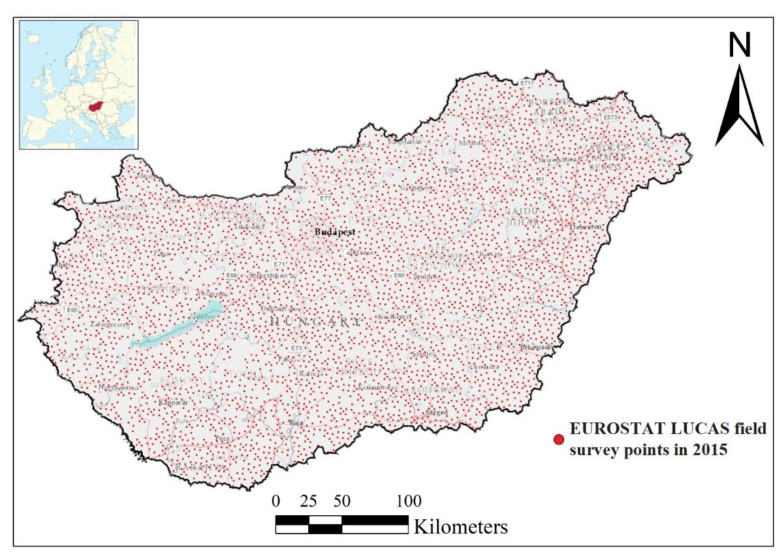
The LUCAS field survey points of Hungary in 2015.

We mapped the occurrence of five widespread invasive plant taxa in Hungary: common milkweed (*A. syriaca),* tree of heaven (*A. altissima*), Russian olive (*E. angustifolia*), black locust (*R. pseudoacacia)* goldenrods (*Solidago* spp.). The LUCAS land cover database is a survey of EUROSTAT, which has provided data on land use/land cover at 330,000 field observation points in 27 European countries every three years since 2009. In 2015, 5169 LUCAS field survey points were surveyed in Hungary. The points were equally distributed over the country with an average distance of 3145 m from each other. At each LUCAS observation point, five field photographs were taken during the field survey: towards the north, south, east and west directions, as well as one of the point itself [26,31]. The visual interpretation of the 2015 LUCAS field survey photos was applied in this study to analyse the spatial distribution of the five investigated invasive plant taxa in country scale. We marked the investigated plants on the LUCAS field photos only in those cases when the individuals of the given plant species were clearly visible. We checked more than 25,845 photos of the 5169 LUCAS observation points from the year 2015 (Figure 1). All of the photos were taken during the vegetation period of 2015. If we could identify at least one of the studied plant species, in at least one photo, we considered that point as invaded with that species [27,32].

As *Solidago canadensis* and *Solidago gigantea* could not be distinguished from each other in most of the photographs, they were identified only at the genus level as *Solidago* spp. As the LUCAS survey points come from a predefined network of equally spaced field observation points, they are more suitable for the monitoring and GIS analyses of biological invasion, than citizen science source datasets, because the crowd source databases are spatially highly fragmented, and do not give realistic spatial information about the distribution of the infected and non-infected areas. For our analyses, the LUCAS points that do not contain the invasive plants are also important information due to the uniform point distribution of the LUCAS data obtained from LUCAS photos.

**Figure 2:**
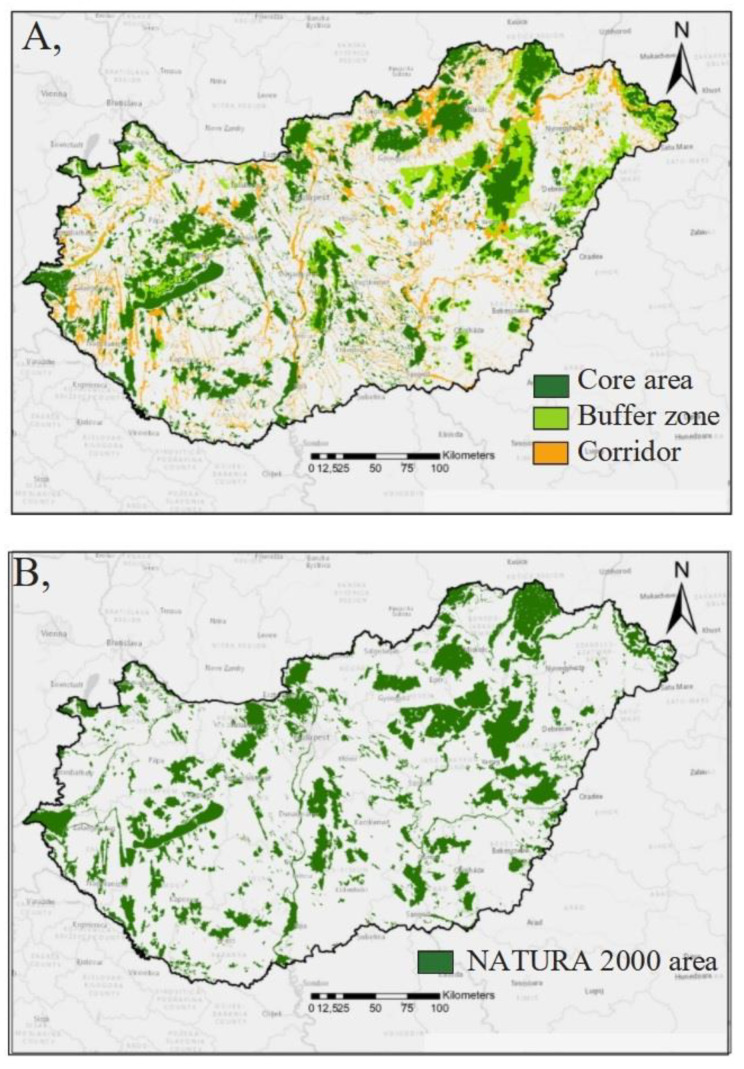
Spatial structure of the HEN (**A**) and Natura 2000 network (**B**) in Hungary.

The digital map of the road and railway networks were obtained from the OpenStreetMap (OSM) 2016 database [33], which contains the first and secondary level paved roads and the railway network for the total territory of Hungary. The digital map of the water network of Hungary (including spatial data of all streams, rivers and artificial channels) was also extracted from the OSM database. The network of Natura 2000 network has been established for the implementation of 2 EU Directives, for wildlife protection, and to improve the migration of valuable plant and animal species [34]. We also used the digital maps of the Natura 2000 network in Hungary [35], and the HEN [36] (Figure 2). The HEN is part of the spatial plan of Hungary and it contains three categories; core area, buffer area and ecological corridor. The core area is the inner area of a biosphere reserve which is legally protected and where only the minimum amount of human activity is allowed. The ecological corridor is a strip of natural habitat surrounded by developed land that connects two or more larger areas of natural habitat (or nature reserves), allowing species to migrate from one site to another. The buffer zone is an area of land usually around a sensitive wildlife habitat, that contains undisturbed vegetation and is designed to minimize sharp changes in habitat or prevent disturbance from surrounding land uses [37].

### 2.3. GIS and Statistical Analyses

Following the identification of infected and non-infected LUCAS points, a spatial database was created using the ArcGIS 10.3 geographic information system based on the geographic coordinates recorded by handheld GPS during field surveys. Using the ArcGIS software, we integrated and overlaid the digital thematic maps of different sources, including the infected and non-infected LUCAS points of the investigated five invasive plant species.

**Figure A1:**
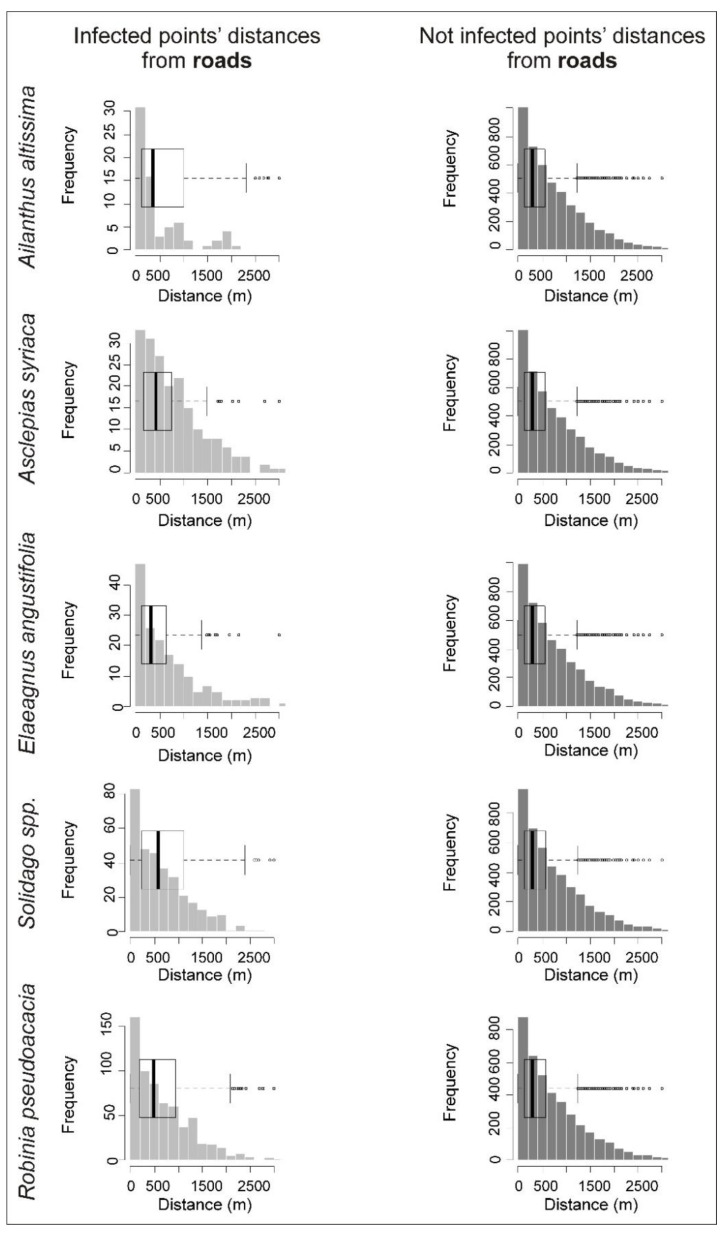
Histogram and boxplots of infected and not infected points’ distances from roads.

**Figure A2:**
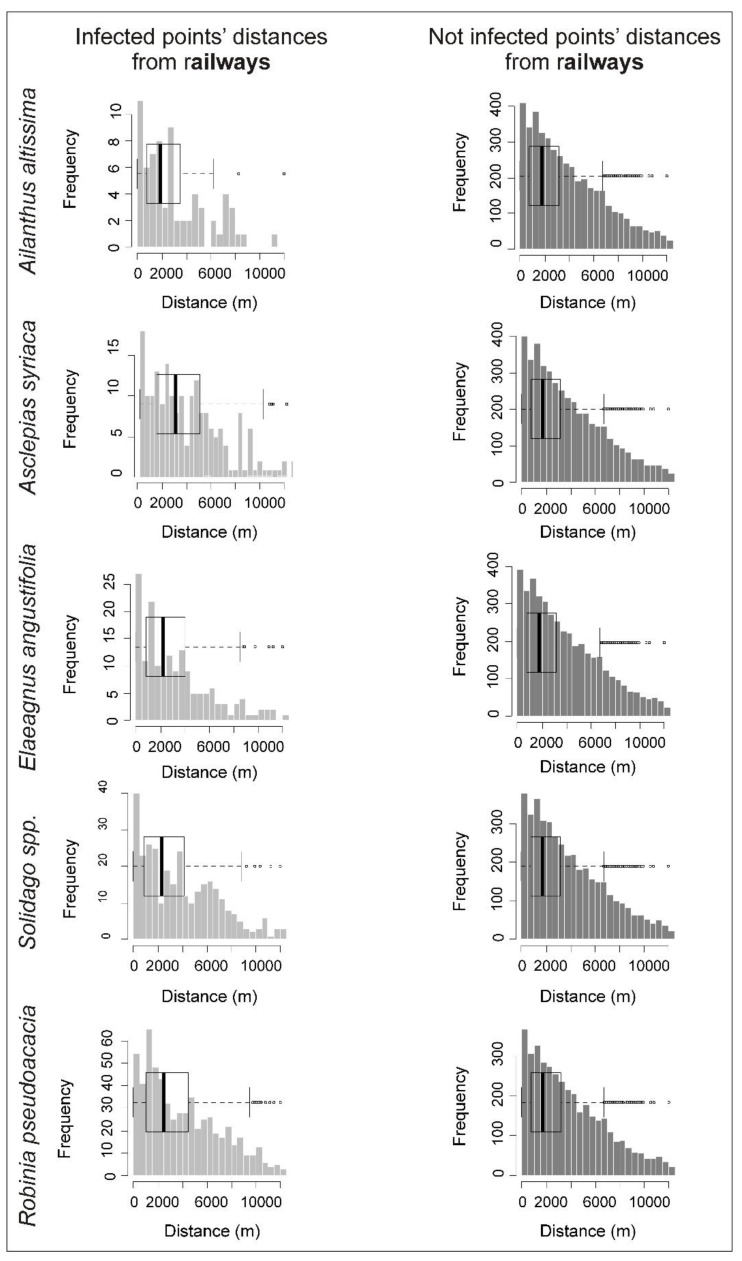
Histogram and boxplots of infected and not infected points’ distances from railways.

**Figure A3:**
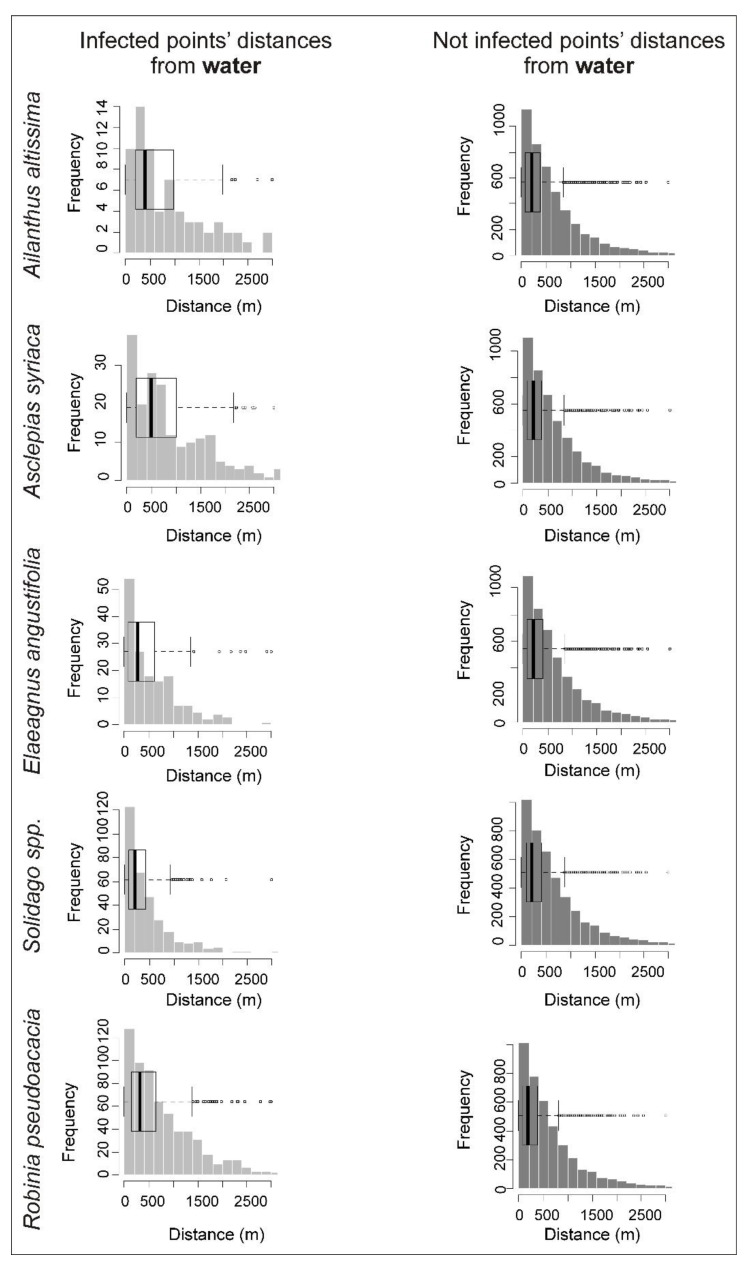
Histogram and boxplots of infected and not infected points’ distances from water.

We calculated the Euclidean distances of each infected and non-infected LUCAS point from the closest element of the road and railway network, and the surface water network maps for all five investigated invasive species by the nearest neighbour tool of the ESRI ArcGis software. To visualize our results, we created histograms and boxplots about the distances in case of the 5 plants species (see in Figure A1, Figure A2 and Figure A3). The graphs have been made in R using the “hist” and “boxplot” functions. Then, we expressed the differences in the mean distances between the infected and non-infected LUCAS points from the linear infrastructures and water network using the following formula:

$$Dist_{a}=\left(\frac{\;\sum_{1}^{n}I\;}{n}\right)-\left(\frac{\;\sum_{1}^{m}\mathrm{NI}\;}{m}\right)$$

where $Dist_{a}$ = the difference in average distances (in metres) between infected and non-infected LUCAS points of a given invasive plant species *a* from the road, railway or water network, *n* = the number of infected LUCAS points, *m* = number of non-infected LUCAS points, *I* = distance of the infected LUCAS points from the road, railway or water network, *NI* = distance of the non-infected LUCAS points from the road, railway or water network.

If the $Dist_{a}$ value is negative (the infected LUCAS points are closer to road, rail- or waterways than the non-infected points), it indicates that the species is more associated with roads, or railway network or green or blue infrastructure. However, if the difference is a positive number, then there is no relationship between linear landscape features and the point-based occurrence data of the plant species.

We calculated the difference in standard deviations of the distances of infected and non-infected points from road, or rail and water networks using the following formula

$$DSD_{a}=\left(\sqrt{\frac{\sum {\left|I-MI\right|}^{2}}{n}}\right)-\left(\sqrt{\frac{\sum {\left|NI-MNI\right|}^{2}}{m}}\right)$$

where $DSD_{a}$ = the difference in standard deviations (in meters) between infected and non-infected LUCAS points of a given invasive plant species *a* from the road, or railway or water network, and *MNI* = the average distances (in metres) between non- infected LUCAS points of a given invasive plant species *a* from the road, railway or water network.

If the $DSD_{a}$ value is negative (the standard deviation of the distances between the point occurrence data and the linear landscape features is smaller than the standard deviation of the mean distances of the non-infected points from the linear landscape features), the infected points are clustered near the linear landscape features, and the species are facilitated by road, or railway, or water networks.

From a conservation point of view, it is important to understand the relationship between the ecological networks and biological invasion. The HEN, which links different protected natural areas, was established in 1993 to improve the migration of native plant and animal species. Most of the core areas are protected areas, and the corridors improve their connectivity. The number of infected and non-infected LUCAS points was summarized inside and outside of the spatial units (core area, buffer area, and ecological corridor) of the HEN, and their percentage distribution was calculated using the following formulas:

$$PI_{eco}=\frac{\sum_{1-3}I_{eco}}{\sum I}*100$$

$$PI_{out}=\frac{\sum I_{out}}{\sum I}*100$$

$$PNI_{eco}=\frac{\sum_{1-3}NI_{eco}}{\sum NI}*100$$

$$PNI_{out}=\frac{\sum NI_{out}}{\sum NI}*100$$

where *PI_eco_* = the proportion in % of LUCAS points infected with one of the species studied within the Hungarian Ecological Network categories.

*PI_out_* = the proportion in % of LUCAS points infected with species infected with one of the species studied outside areas of the Hungarian Ecological Network categories.

*PNI_eco_* = the proportion in % of LUCAS points non-infected with species among the five species surveyed within the Hungarian Ecological Network categories.

*PNI_out_* = the proportion in % of LUCAS points non-infected with species among the five species surveyed outside areas of the Hungarian l Ecological Network categories.

*I* = number of all LUCAS points infected with a given species.

*I_eco_* = number of all LUCAS points infected with a given species inside a given category from the three HEN categories.

*NI* = number of all LUCAS points not infected with a given species.

*NI_eco_* = number of all LUCAS points not infected with a given species inside given category from the three HEN categories.

*NI_out_* = number of all LUCAS points not infected with a given species outside areas of the Hungarian Ecological Network categories.

The number of infected and non-infected LUCAS points was also calculated inside and outside of the Natura 2000 network of Hungary using the following formula:

$$P_{nat}=\frac{\sum I_{nat}}{\sum I}*100$$

$$P_{onat}=\frac{\sum I_{onat}}{\sum I}*100$$

$$PNI_{nat}=\frac{\sum_{1-3}NI_{nat}}{\sum NI}*100$$

$$PNI_{onat}=\frac{\sum NI_{onat}}{\sum NI}*100$$

where *PI_nat_* = the proportion in % of LUCAS points infected with species among the five species surveyed within the Natura 2000 areas of Hungary.

*PI_onat_* = the proportion in % of LUCAS points infected with species among the five species surveyed outside of the Natura 2000 areas of Hungary.

*PNI_nat_* = the proportion in % of LUCAS points non-infected with species among the five species surveyed within the Natura 2000 areas of Hungary.

*PNI_onat_* = the proportion in % of LUCAS points non-infected with species among the five species surveyed outside areas of the Natura 2000 areas of Hungary.

*I* = number of all LUCAS points infected with a given species.

*I_nat_* = number of all LUCAS points infected with a given species inside of the Natura 2000 areas of Hungary.

*NI* = number of all LUCAS points not infected with a given species.

*NI_nat_* = number of all LUCAS points not infected with a given species inside of the Natura 2000 areas of Hungary.

*NI_onat_* = number of all LUCAS points not infected with a given species outside of the Natura 2000 areas of Hungary

## 3. Results

Based on field photographs in the LUCAS database, digital point maps of species occurrence were prepared for each of the five invasive plants. The number of infected points for each species was much lower than the number of non-infected points, but sufficient to analyse the spatial relationship between infection and distance to roads, or railways or water networks (Table 1). See the histograms and boxplots of these distances in the Appendix A (Figure A1, Figure A2 and Figure A3).

**Figure 3:**
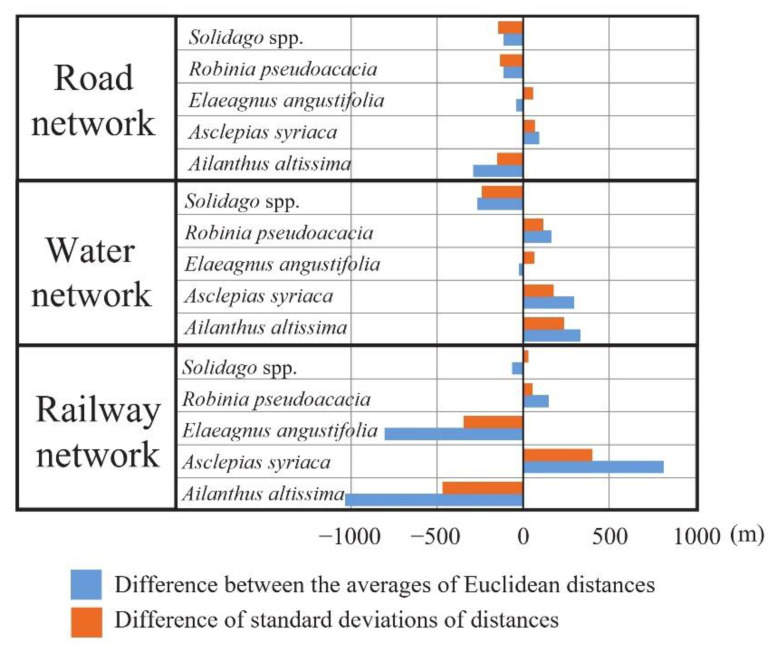
Difference in average and standard deviation of Euclidean distances between infected and non-infected LUCAS points and roads, railway networks, and green and blue infrastructure.

If the average distance of points infected with the invasive species under study from the investigated linear landscape features is less than the average distance of non-infected LUCAS points, then its difference is negative (Figure 3).

We found that for plants with larger differences in the mean distances of infected and non-infected points by linear elements, the standard deviations of the Euclidean distance data also show significant differences. For almost all the studied plants, the differences in the mean distances of their infected and uninfected points from roads, or railway networks, or green and blue infrastructure, and the differences in the distance data and standard deviations have the same sign.

Of the five investigated invasive species, LUCAS points infected with *A. altissima* are on average 1035 m closer to the rail network than non-infected points. Furthermore, for *E. angustifolia*, the difference between the average distance between infected and non-infected is close to 1000 m (838 m). The standard deviation of the distances from the railway network of the points infected by these two species is also nearly 500 m smaller than that from the railway network of the non-infected LUCAS points. Thus, these two species are clearly more frequently occurring along railway lines, whereas the occurrence data for *A. syriaca* and *R. pseudoacacia* are not affected by the railway network, as the LUCAS points infected by them are on average at or closer to the railway network than the non-infected points. In the case of goldenrod, there is no clear correlation between the railway network and the occurrence of the plant.

On average, LUCAS points infected with goldenrod are 264 m closer to the water network (streams, canals, and rivers) than non-infected points. The standard deviation of the distances of these infected LUCAS points from water networks is also much smaller (239 m) than from non-infected points. Goldenrod is therefore clearly a characteristic plant in floodplain habitats, and its appearance can be linked to wetland habitats. Regarding the other investigated species, only points infected with *E. angustifolia* are closer (24 m) to the water network than the uninfected points. The occurrence of black locust and common milkweed does not show any relationship with the blue infrastructure, as the average distance of infected points from water network elements (streams, canals and rivers) is greater than the average distance of non-infected points.

The proximity of paved roads has a detectable effect on three of the five invasive species (*A. altissima*, *Solidago* spp., *R. pseudoacacia*). For all three species, the distance between infected LUCAS points and the road network is smaller than that of non-infected points, therefore the road network plays an important role in their occurrence and spread. Although the LUCAS points where *E. angustifolia* occurs are on average 39 m closer to the road network than the non-infected points, the standard deviation of distance data for infected points is larger than for non-infected points, and therefore it is not clear whether there is a relationship with the road network. In addition, points infected with *A. syriaca* are on average further away from the road network than LUCAS points not infected with common milkweed.

Among the investigated invasive plant species, LUCAS points infected by *Solidago* spp. were overrepresented compared to non-infected points in all spatial units (buffer area, core area and ecological corridor) of the HEN (Table 2). 

Our results show that ecological corridors provide a pathway for the spread of invasive plant species, as LUCAS points infected by all the investigated plants except *A. syriaca* and *E. angustifolia* are more prevalent than non-infected points within ecological corridors. Surprisingly, within the core area of the HEN, only *A. syriaca* has a lower infection rate than non-infected points. This suggests that highly protected nature conservation areas (e.g., national parks) are more infected with *A. altissima*, *Solidago* spp., and *R. pseudoacacia* than areas outside protected areas (Table 2).

Within Natura 2000 sites, the occurrence of *Solidago* spp. and *E. angustifolia* species infected LUCAS points is overrepresented compared to non-infected LUCAS points (Table 3).

## 4. Discussion

Our results show that the Hungarian railway network (embankments) may play an important role in the spread of *A. altissima* and *E. angustifolia*. One of the main reasons for this pattern would be that these highly drought-tolerant species have an advantage on the dry, compacted and contaminated soils of railway embankments and in other highly disturbed areas compared to many other species [38,39].

On the other hand, the passing trains generate winds (secondary winds) that may help the spread of certain invasive species [40]. Along the road network, the occurrence of *E. angustifolia*, *Solidago* spp. and *R. pseudoacacia* was more common than in other areas. The intensive spread of *Solidago* spp., *A. altissima* and *R. pseudoacacia* along roadsides is confirmed by the international literature [19,40]. In contrast to the other species, *R. pseudoacacia* is often deliberately planted along roads, while the other mentioned species (*E. angustifolia*, *Solidago* spp. and *A. altissima*) are not, and it seems evident that the road network is facilitating their spontaneous spread (presumably with the help of secondary winds generated by traffic).

The role of railway and road networks in the spread of invasive plants is somewhat different, although the main driving force (i.e., secondary wind dispersal) behind species invasion would be the same [20,40]. However, railway and road networks may differ in some essential conditions. The railway network is typically flanked by embankments, which provide dry habitats, whereas the roads are flanked by drainage ditches, which provide typical wetland habitats. 

Our results do not support the finding of Kowarik and Säumel [18] that the reproduction of the *A. altissima* is influenced by the water network. This may explain why some invasive species associated with high moisture conditions (e.g., *Solidago* spp.) are more common along roads and are rare or absent from railway habitats. According to Follak et al. [19] and Vorstenbosch et al. [21], the occurrence of *A. syriaca* in Austria is more frequent along roads than in many other habitats. However, other authors have noted that this relationship is strongly dependent on the type and structure of surrounding landscape [20]. This can be explained by the fact that the elevation, landscape structure (configuration and composition) and land use of Austria’s mountainous landscapes are very different from those of Hungary. We also demonstrated that the distribution of *Solidago* spp. is often related to roadsides, railway embankments, and streams [1,41], and that many habitats within the Natura 2000 and national ecological networks are heavily infected with invasive species.

This indicates that biological invasions are already affecting not only ecological corridors but also core areas with high ecological sensitivity in Hungary [16], similar to other European countries [2,3,42,43,44]. Therefore, targeted management practices (e.g., mowing, grazing, and mechanical control) are required to suppress the populations of invasive species and to maintain ecosystem services within protected areas [45,46,47]. In some cases, the designation of ecological corridors needs to be re-designed.

Using the LUCAS database to estimate the distribution of various invasive species and their impact on the biota of natural and semi-natural habitats may be a cost-effective alternative for the detection of these species. In recent years, several papers have been published on the advantages and drawbacks of photograph-based techniques in relation to the distribution of different species, such as Google Street View [48,49,50,51]). These papers conclude that virtual survey is cost-effective, allows the handling of a large volume of data and may perform well in the identification of many species at least to the genus level. However, its efficiency is variable across seasons and strongly depends on the density of data points. In addition, these methods often fail to capture small-scale patterns, such as the exact number of individuals and special traits of species (e.g., diameter of trees, plant height and number of flowers per individual). Similar conclusions can be drawn when considering the LUCAS database for estimating the rate of plant invasion across different habitats.

## 5. Conclusions

In our study, we developed a GIS methodology for quantitative analysis of the relationship between roads, railway networks, or green and blue infrastructure and biological invasion. Maps of invasive plants based on an equal distance field survey observation point network (LUCAS points) are suitable for analysing the relationship between linear infrastructure and the occurrence or absence of the invasive plant species. In our study, we have shown that roads, railways, water and ecological networks play an important role in the occurrence and spread of certain invasive plants in Hungary. Using GIS based quantitative methods, we identified and verified which of the five studied common invasive plant species occur more frequently along linear landscape features, blue infrastructure, and in Natura 2000 areas and ecological networks in Hungary. It has been shown that the occurrence of certain invasive plants (e.g., *A. syriaca*) along the road network is strongly dependent on the structure of the roadside landscape and dominant land use. For instance, in the Hungarian agricultural (arable land dominated) landscapes, the occurrence of this species is not related to the road network. Conversely, the occurrence of other species (e.g., *A. altissima*) is overrepresented along linear infrastructure (roads and railways) regardless of the type and structure of the surrounding landscape. According to our findings, ecological networks, and Natura 2000 areas can support the invasion of some invasive plant species (*Solidago spp.* and *E. angustifolia*).

The presented method may be suitable for identifying, mapping, and monitoring invaded areas, and the occurrences of certain invasive plant species. The obtained results can be used to model invasion risk, to plan land use (e.g., canal network, roads, and railways) and to help improve management priorities for the ecological network of the protected areas. Our results are useful for modelling and predicting the spread of invasive plant species, its early detection [52] and to select those variables which would be important input data for spreading models and for further biological invasion vulnerability maps at regional scale.

## Figures and Tables

**Table 1:** Number and proportion of invasive plants surveyed at LUCAS field monitoring points.

Invasive Species	Number of Infected LUCAS Points	Proportions of the Infected LUCAS Points (where 100% = 5169)
*Ailanthus altissima* (Mill.) Swingle	71	1.4%
*Asclepias syriaca* L.	195	3.8%
*Elaeagnus angustifolia* L.	168	3.3%
*Solidago* spp.	324	6.3%
*Robinia pseudoacacia* L.	630	12.2%

**Table 2:** The spatial distributions of LUCAS points infected and non-infected by the invasive plants, inside and outside the spatial units of the HEN (The bold numbers show those proportion data-pairs, where the proportion of a given invasion plant infected points is higher (overrepresented), than the proportion of non-infected LUCAS points within the given territorial unit).

Invasive Species	Type of the LUCAS Points	Out of the HEN (%)	Spatial Units of the HEN				Total (%)
			Core Area (%)	Buffer Zone (%)	Corridor (%)	Subtotal (%)	
*Ailanthus altissima*	Infected Non-infected	70.4 72.5	**12.7** **12.5**	**8.5** **7.1**	**8.5** **7.9**	29.6	100
						27.5	100
*Asclepias syriaca*	Infected Non-infected	**80.5** **72.2**	9.7 12.6	2.6 7.3	7.2 7.9	19.2	100
						27.8	100
*Elaeagnus angustifolia*	Infected Non-infected	63.1 72.8	**19.6** **12.3**	4.8 7.22	**12.5** **7.7**	36.9	100
						27.2	100
*Robinia pseudoacacia*	Infected Non-infected	**72.5** **72.4**	**13.2** **7.8**	6.9 8.6	7.4 11.3	27.5	100
						27.6	100
*Solidago* spp.	Infected Non-infected	63.3 73.1	**14.5** **12.4**	**8.6** **7**	**13.6** **7.5**	36.7	100
						26.9	100

**Table 3:** The spatial distribution of LUCAS points infected and non-infected by the invasive plants, inside and outside Natura 2000 areas in Hungary (The **bold numbers** show the results where the proportion of the given invasion plant (LUCAS point) is higher (overrepresented) than the proportion of non-infected LUCAS points within the given unit).

Invasive Species	Type of the LUCAS Points	Out of the Natura 2000 Areas (%)	Natura 2000 Areas (%)	Total (%)
*Ailanthus altissima*	Infected Non-infected	**93** **85.1**	7 14.9	100
				100
*Asclepias syriaca*	Infected Non-infected	**94.4** **84.8**	5.6 15.2	100
				100
*Elaeagnus angustifolia*	Infected Non-infected	78.6 85.4	**21.4** **14.6**	100
				100
*Robinia pseudoacacia*	Infected Non-infected	**90.8** **84.4**	9.2 15.6	100
				100
*Solidago* spp.	Infected Non-infected	83.6 85.3	**16.4** **14.7**	100
				100

## Data Availability

The spatial occurrence data of the five investigated invasive plants is available on the web map of the University of Szeged, Department of Geoinformatics, Physical and Environmental Geography, “National GIS Database of Invasive Plant Species”: http://www.geo.u-szeged.hu/invasive/index_en.html, accessed on 1 December 2021.
